# Acute Response of Peripheral Blood Cell to Autologous Hematopoietic Stem Cell Transplantation in Type 1 Diabetic Patient

**DOI:** 10.1371/journal.pone.0031887

**Published:** 2012-02-22

**Authors:** Xiaofang Zhang, Lei Ye, Jiong Hu, Wei Tang, Ruixin Liu, Minglan Yang, Jie Hong, Weiqing Wang, Guang Ning, Weiqiong Gu

**Affiliations:** 1 Shanghai Key Laboratory for Endocrine Tumors, Shanghai Clinical Center for Endocrine and Metabolic Diseases, Shanghai Institute of Endocrine and Metabolic Diseases, Shanghai E-institute for Endocrinology, School of Medicine, Shanghai Jiaotong University, Ruijin Hospital, Shanghai, People's Republic of China; 2 Laboratory for Endocrine and Metabolic Diseases, Institute of Health Science, Shanghai Institutes for Biological Sciences, School of Medicine, Shanghai JiaoTong University, Chinese Academy of Sciences, Shanghai, People's Republic of China; 3 Department of Hematology, School of Medicine, Shanghai Jiaotong University, Ruijin Hospital, Shanghai, People's Republic of China; The University of Hong Kong, Hong Kong

## Abstract

**Objective:**

Autologous nonmyeloablative hematopoietic stem cell transplantation (AHST) was the first therapeutic approach that can improve β cell function in type 1 diabetic (T1D) patients. This study was designed to investigate the potential mechanisms involved.

**Design and methods:**

We applied AHST to nine T1D patients diagnosed within six months and analyzed the acute responses in peripheral blood for lymphocyte subpopulation as well as for genomic expression profiling at the six-month follow-up.

**Results:**

We found six patients obtained insulin free (IF group) and three remained insulin dependent (ID group); C-peptide production was significantly higher in IF group compared to ID group. The acute responses in lymphocytes at six-month follow-up include declined CD3^+^CD4^+^, CD3^+^CD8^+^ T cell population and recovered B cell, NK cell population in both groups but with no significant differences between the two groups; most immune-related genes and pathways were up-regulated in peripheral blood mononuclear cell (PBMC) of both groups while none of transcription factors for immune regulatory component were significantly changed; the IF group demonstrated more AHST-modified genetic events than the ID group and distinct pattern of top pathways, co-expression network as well as ‘hub’ genes (eg, TCF7 and GZMA) were associated with each group.

**Conclusions:**

AHST could improve the islet function in newly diagnosed T1D patients and elimination of the islet specific autoreactive T cells might be one of the mechanisms involved; T1D patients responded differently to AHST possibly due to the distinct transcriptional events occurring in PBMC.

**Trial Registration:**

ClinicalTrials.gov NCT00807651

## Introduction

Type 1 diabetes (T1DM) is an organ specific autoimmune disease, resulting from chronic immune attack against pancreatic beta cells [1]. Although it is thought to be mediated mainly by T helper 1 cells, a complex interaction of immune cells including CD4^+^T cell, CD8^+^Tcell and innate immune cell NK cell, B cell and antigen presentation cell is actually involved in the pathogenesis [2]. This course of immune-destruction is subclinical until approximately 60% to 80% of the beta-cell mass is destroyed, when the amount of beta-cell mass is insufficient to maintain glucose homeostasis and the clinical diagnosis of T1DM is established [3]. The best-established treatment is to tightly control the blood glucose by intensive insulin therapy [4]. However, long-term substitutive insulin therapy is still associated with major constraints and lack of effectiveness in preventing chronic vascular and neurological complications. Immunointervention therapy, which targets the causal pathogenic mechanism, therefore, may represent the only sensible strategy. Clinical trial of immunosuppression drugs (cyclosporine), antigen therapy (GAD) or immunoregulatory agents (anti-CD3 antibody) have obtained efficacy in patients with T1DM, however obstacles such as adverse effects, lack of long-lasting improvement and especially the exogenous insulin requirement remain still as a medical challenging [5].

In 2007, Julio C. Voltarelli's group reported the first clinical trial using AHST as a potential approach in cases of T1D [6]. Indeed, for the first time these studies demonstrated that AHST led to prolonged insulin independence coupled with a significant increase of c-peptide production. A follow-up study published two years later confirmed the insulin independence was due to improved β cell function instead of a prolonged honeymoon [7]. Therefore, AHST has been the only T1D-related management shown to preserve β cell function. However, AHST requires a relatively aggressive immune-intervention and complications such as pneumonia and endocrine dysfunction have been noted. Therefore, a need exists to be able to target those patients who will receive the most benefit from AHST and to further clarify the mechanisms involved in β cell function recovery so that the strategy can be optimized and a broader patient population can benefit from this treatment approach.

In this study, we applied AHST therapy to a group of nine patients with newly diagnosed T1D and specifically investigated their immune reconstitution, as well as performed transcriptome profiling on their PBMC pre-treatment and six months post-treatment to identify the acute responsive events, which might give helpful insights to clarify the therapeutic mechanisms.

## Methods

### Patient management

Nine subjects diagnosed as T1D within six months by clinical findings and hyperglycemia and confirmed with positive antibodies against glutamic acid decarboxylase (GAD) [8] were enrolled from January 2009 to December 2009 in our hospital (Table 1). Patients with history of ketoacidosis onset, presence of acute or chronic infection, possibility of pregnancy or any organ dysfunction were excluded from this study. Patients were put on an intensive insulin therapy to maintain normoglycemia until transplantation. Transplantation was performed on average 2.0±0.9 months following initial diagnosis as previously reported [6]. Briefly, hematopoietic stem cells were mobilized with cyclophosphamide (2.0 g/m^2^) and granulocyte colony stimulating factor (10 µg/kg per day) and then collected from peripheral blood by leukapheresis and cryopreserved. The cells were injected intravenously after conditioning with cyclophosphamide (200 mg/kg) and rabbit antithymocyte globulin (4.5 mg/kg). Number of infused CD34^+^ cells was displayed in Table 1. The patients were followed up for glucose level, HbA1c, C-peptide level, GAD antibody and insulin dosage. All patients provided written informed consent in accordance with the protocol approved by the board of medical ethics of Ruijin Hospital. The board of medical ethics of Ruijin Hospital approved our study and our study complied with the request of the board of medical ethics of Ruijin hospital.

**Table 1:** Pretreatment and follow-up variables of patients with type 1 diabetic mellitus undergoing autologous nonmyeloablative hematopoietic stem cell transplantation.

					Pre-treatment				Insulin Dose (IU/Kg/day)			C-peptide^g^		
Case/sex	Age(Y)	BMI(kg/m^2^)	Stem cell Infusion (CD34^+^*10^6^/Kg)	Duration(mo)^a^	FBG^b^	PBG^c^	HbA1c^d^	GADA^e^	pre-treatment	6 mo	12 mo	pre-treatment	6 mo	12 mo
1/F	18	17.2	5.95	2.5	6.2	13.8	7.8	410	0.62	0	0	0.11	1.05	1.57
2/M	17	18.7	10.8	1	9.7	9.9	12.9	41.7	0.91	0	0	0.62	1.26	1.25
3/F	21	17.7	17.17	1.5	6.7	8.9	14	153	0.85	0	0	0.62	1.17	1.07
4/M	25	20	8.96	2	7.3	11.3	8.8	289.1	0.41	0.07	0	0.53	1.12	1.58
6/F	15	17.6	24.95	4	6.1	10.5	7.1	2238	0.43	0	0	0.76	0.96	1
9/F	15	16.4	11.49	1.5	5.7	4.6	11.5	4050	0.72	0	0	0.69	1.23	-
Mean	18.5	17.9	13.22	2.1	7	9.8	10.4	864.8	0.66	0.01	0	0.56	1.13	1.29
5/M	15	17.9	7.5	2	4.1	12.1	9.2	60.2	0.61	0.11	0.46	0.4	0.23	0.39
7/M	14	20.6	12.3	1.5	6.5	11.1	11.1	49.1	0.57	0.25	0.5	0.39	0.42	0.58
8/M	18	20.6	11.68	2	4.4	9.4	9.2	146.7	0.56	0.48	0.19	0.46	1.44	0.85
Mean	15.7	19.7	10.49	1.8	5	10.9	9.8	85.3	0.58	0.28	0.38	0.42	0.7	0.61
P^f^	0.3	0.083	1	1	0.167	0.714	1	0.167	0.548	0.012	0.012	0.143	0.083	0.036

a Duration referred to duration time from appearance of symptoms of hyperglycemia to treatment of AHST (mo);

b FBG (mmol/L);

c PBG(mmol/L);

d HbA1c (%);

e GADA (U/mL);

f P value, comparison between IF group (patient 1, 2, 3, 4, 6, 9) and ID group (patient 5, 7, 8) using Mann-Whitney test;

g C-peptide (ng/mL); - represent the data did not been acquired.

### Flow cytometry

Blood collections were performed prior to stem cell mobilization to establish a baseline, and at six months post-treatment. Measurements of forward and side scatter were combined with CD45 to identify lymphocytes and exclude monocytes. Absolute subpopulations of lymphocyte numbers were calculated based on the total lymphocyte counts and the percentage of subpopulations of lymphocyte cells, as identified by flow cytometry using the BD Multitest panel (BD Biosciences). The fraction of lymphocyte cell subsets was determined by four-colour FACS analysis using appropriate surface markers: anti-CD3-PC5; anti-CD45-FITC; anti-CD4-RD1; anti-CD8-ECD and anti-CD45-PC5; anti-CD19-FITC; anti-CD20-FITC and anti-CD45-PC5; anti-CD16-PE; anti-56-PE; anti-CD3-FITC (Beckman, UK).

### Microarray experiment

Comparative microarray profiling was performed pre-treatment and at six months post-transplantation. PBMC was isolated within six hours by Lymphoprep^Tm^ gradient purification according to the manufacturer's instructions (Axis-Shield PoC AS, Oslo, Norway) and stored in liquid nitrogen until use. Total RNA was isolated by RNAEasy (Qiagen, Valencia, CA) and RIN values assessed using an Agilent 2100 Bioanalyzer. cDNA synthesis, hybridization, and staining were performed as specified by Affymetrix Human Genome U133 Plus 2.0 Arrays, using an automated GeneChip Fluidics Station 450 and Affymetrix Scanner 3000 7 G to generate CEL files (Santa Clara, CA). All data is MIAME compliant and the raw data has been deposited in GEO database (GSE29908).

### Data analysis

Data analysis was performed with the assistance of Genminix Informatics Ltd., Co (Shanghai). Gene expression levels were normalized using the robust multiarray average (RMA) procedure and then were normalized using the median normalization method for differential genes. Differentially expressed genes (pre-treatment vs post-treatment) were selected by software R and filtered by random variance model (RVM) [9] paired T test when p<0.05 and FDR<0.05. Pathway analysis was performed using the BioCarta database (www.biocarta.com) and computed by Fisher's exact test and Chi-square test to identify pathways modified by AHST when p<0.05 and FDR<0.05. For the differential co-expression network [10], we first created a gene-set by combining the differently expressed genes showing a response to AHST in the IF and ID groups. Secondly, we merged the six samples in IF group into three based on the similarity in expression profile of individual sample. Co-expression networks were generated by calculating the coefficient correlation of the combined gene-set in the four data sets: pre-treatment in IF, post-treatment in IF, pre-treatment in ID and post-treatment in ID group (Figure S1). We defined a connectivity measure (K) for each gene based on its Pearson correlation with all of the other genes in each network and divided by the maximum network connectivity. The differential connectivity of pre-treatment and post-treatment was defined as DiffK = K(post-treatment)-K(pre-treatment). The ‘hub gene’ was identified when DiffK>0.2 or <−0.2 and p<0.05.

### Statistical analysis

The logarithm (base e) of GADA was proposed when statistical analysis was performed. A paired T test was used to compare (per patient) pre-treatment and post-treatment data using SPSS statistics 17.0. Significant differences between patient groups were assessed with the Mann-Whitney test (SPSS statistics 17.0). All P values were 2-sided and exact test; statistical significance was set at p = 0.05.

## Results

### Evaluation of clinical response at 12-month follow-up revealed two categories

**Figure 1:**
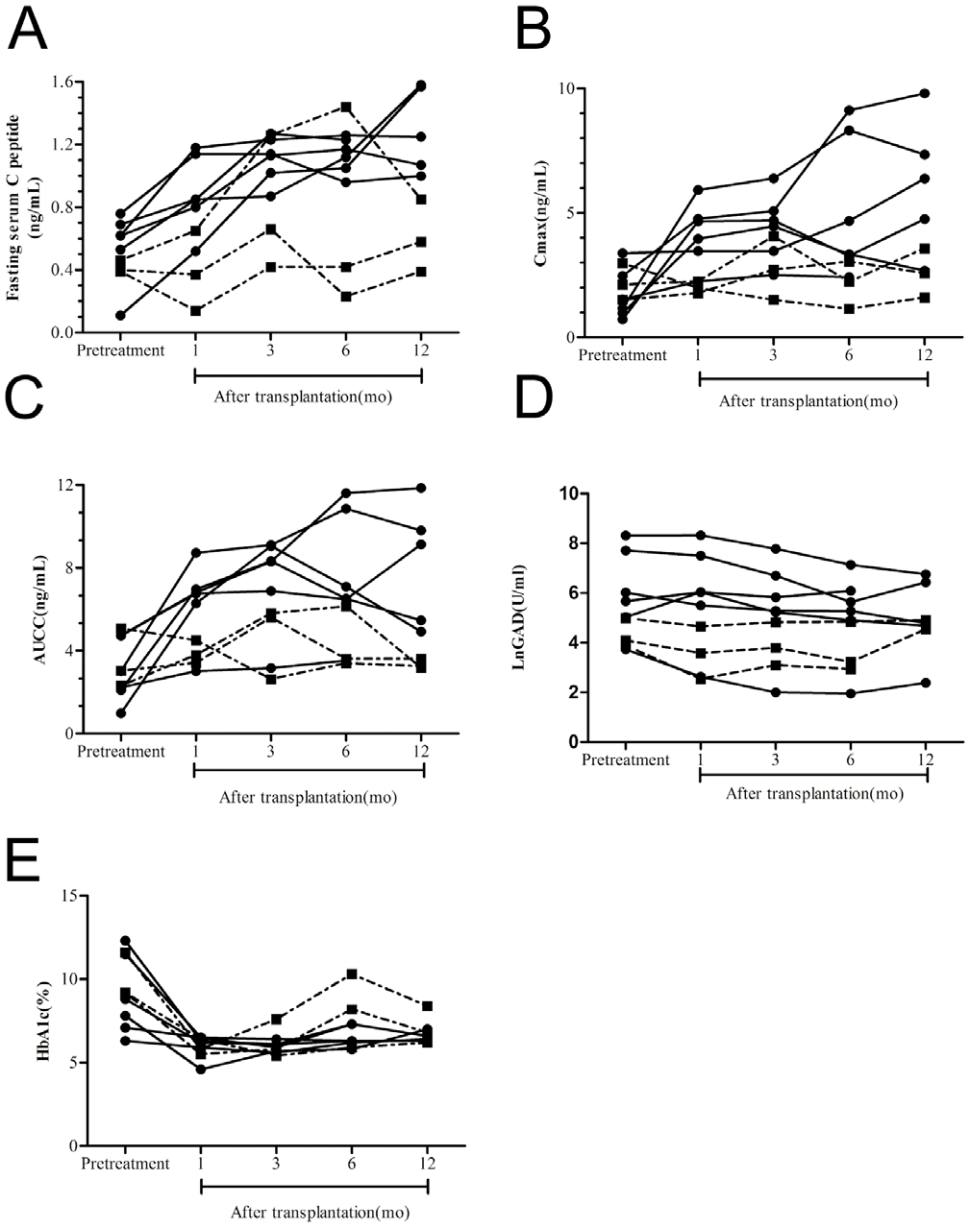
Time course of fasting C-peptide (A), Cmax (B), AUCC (C), GADA (D) and HbA1c (E) in IF group and ID group respectively. Black circles, insulin free. Black squares, insulin dependent. X axis represents the time course relative to HSCT. **A** p<0.05, pre-treatment vs all follow-up time in IF group; IF vs ID group at 1 month and 12 months post-treatment. **B** p<0.05, pre-treatment vs all follow-up times in IF group; IF vs ID group at one month and six months post-treatment. **C** p<0.05, pre-treatment vs all follow-up times in IF group; IF vs ID group at 12 months post-treatment. **D** p = 0.036, IF vs ID group at six months post-treatment.

Within 12-month follow-up period, six patients (1, 2, 3, 4, 6 and 9) became insulin free (defined as IF group), while the remaining patients (5, 7, and 8) still required insulin injection (defined as ID group), although with reduced dosage (average of 0.38 IU/kg/day) (Table 1). For the IF group, the C-peptide production represented by fasting C-peptide, Cmax and AUCC was significantly increased at 1, 3, and 12 months after HSCT (all p<0.05, compared with pre-treatment), while no significant changes were detected in ID group. Furthermore, IF groups showed significant higher levels of fasting C-peptide, Cmax and AUCC at different follow-up time points after transplantation (Figure 1A, B and C). These differences in C-peptide production were not apparent prior to transplantation (Table 1). The HbA1c level decreased significantly one month after transplantation and maintained at less than 7% during 12 months follow-up in all patients except patient 7 in ID group showed HbA1c level at 8.3% at 12 months with poor glucose control (Figure 1E).

At six months follow-up, eight of the nine patients showed declined GADA titers and increased GADA was only noticed in one patient from the IF group. However, there was no significant difference pre-treatment and post-transplantation. Interestingly, the GADA level was significantly decreased in ID group comparing to IF group at six months follow-up (P = 0.036) (Figure 1D).

In comparing the pretreatment parameters of the two groups there were no significant differences related to age, BMI, duration time, glucose levels, HbA1c or GAD antibody (Table 1).

As to the side effects, we noted bacteria and fungus infections only in conditioning time and shortly after AHST treatment: staphylococcus and streptococcus infection in patient 6, 7, 8 and 9; vulvovaginal candidiasis in patient 9. No virus infection was observed. No late complications, such as late infection (>100 days), autoimmune disease and dysfunction of gonad axis have been noticed until 12 months follow-up.

### Lymphocyte subpopulation was distributed independently of insulin use

To examine if acute response in lymphocytes was able to identify a potential mechanism of therapeutic response, we profiled the lymphocyte subpopulation and immune reconstitution in patients at six months. As shown in Table 2, both groups showed declined CD3^+^CD4^+^ T cell populations and recovered CD19, CD20 as well as NK cell counts (CD3^−^CD16^+^CD56^+^) at 6-month follow-up; CD19 and CD20 positive lymphocyte tended to increase in IF group and decrease in ID group; for CD3^+^CD8^+^ T cell, the trend of decline exists in both groups although no statistic significance (p = 0.12) was found, which should be explained carefully because of the small sample size. No significant differences in cell populations were observed between the IF and ID groups either pre-treatment or six months post-treatment (Table 3).

**Table 2:** Phenotype analysis of lymphocyte populations after hematopoieticstem cell transplantation (AHST)^a^.

	IF group			ID group		
Lymphocyte population	At diagnosis	six months after AHST	P value^b^	At diagnosis	six months after AHST	P value^c^
Total	1846±899	926±172	0.05	2179±344	1106±223	0.02
CD3+	1566±769	579±120	0.17	1625±240	644±168	0.01
CD3+CD4+	873±365	186±28	0.009	845±171	230±74	0.03
CD3+CD8+	541±372	318±131	0.12	654±124	353±92	0.004
CD19+	204±134	252±115	0.63	323±39	287±58	0.58
CD20+	206±143	375±302	0.28	335±39	261±72	0.33
CD3−CD16+CD56+	54±47	77±76	0.54	146±95	165±60	0.48

a Values are the mean±SD counts/µL. See Results for description of groups;

b P value between at diagnosis and six months in IF group using paired T test;

c P value between at diagnosis and six months in ID group using paired T test.

**Table 3:** Phenotype analysis of lymphocyte subpopulations at diagnosis and six months after AHST^a^.

	At diagnosis			six months after AHST		
Lymphocyte population	IF	ID	P value^b^	IF	ID	P value^c^
Total	1846±899	2179±344	0.99	926±172	1106±223	0.38
CD3+	1566±769	1625±240	0.91	579±120	644±168	0.71
CD3+CD4+	873±365	845±171	0.92	186±28	230±74	0.25
CD3+CD8+	541±372	654±124	0.91	318±131	353±92	0.70
CD19	204±134	323±39	0.19	252±115	287±58	0.61
CD20	206±143	335±39	0.16	375±302	261±72	0.57
CD3−CD16+CD56+	54±47	146±95	0.10	77±76	165±60	0.11

a Values are the mean±SD counts/µL. See Results for description of groups;

b P value at diagnosis between IF group and ID group using Mann-Whitney test;

c P value at six months after AHST between IF group and ID group using Mann-Whitney test.

### An array-based genomic study revealed transcriptional events associated with insulin dependence

**Figure 2:**
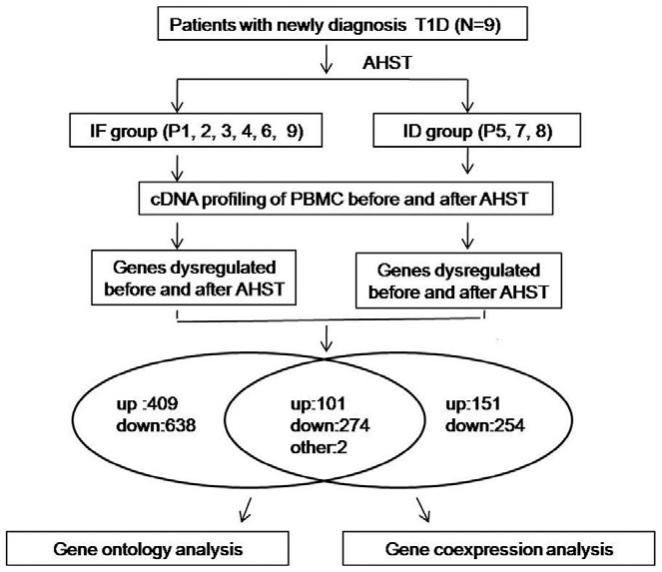
Workflow of the genomic expression profiling of the PMBC in patients with type 1 diabetes. Differentially expressed genes (pre-treatment vs post-treatment) meet the criterion p<0.05 and FDR<0.05. Up, genes with higher expression after AHST. Down, genes with lower expression after AHST. Other, genes that were up-regulated in IF group and down-regulated in ID group.

**Figure 3:**
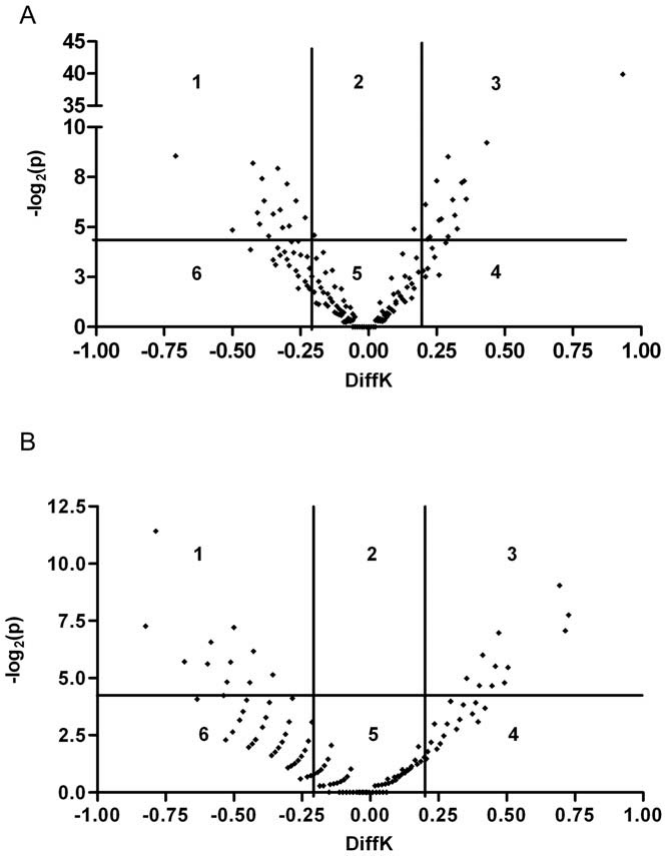
Sector plots of differential network analysis in IF group (A) and ID group (B). The difference in connectivity (DiffK) is plotted on the X axis, and p values are plotted on the Y axis. Horizontal lines indicate a difference in connectivity of −0.2 and 0.2, whereas vertical lines depict a p value 0.05. Number indicates sector 1–6 and plots represent genes. The genes ploted in the sector 1 and 3 are considered as difference in connectivity.

Given the limited signature that can be obtained by lymphocyte population profiling we opted to additionally perform transcriptome analysis. For both the responding (IF) and non-responding (ID) patient groups, we observed that most immune-related genes were up-regulated (Table S1) and the number of significantly regulated genes was greater in the IF group compared to ID group (Figure 2). A majority of the pathway categories were observed in both IF and ID groups, but group-specific pathways also existed. These findings are summarized in Table S2, which shows the top 20 pathways commonly and differentially modified in each group. Top two pathway categories commonly identified in the two groups included (1) selective expression of chemokine receptors during T-cell polarization and (2) IL12 and Stat4 dependent signaling pathway in Th1 development. These two pathways indicated that T cell was differentiated to Th1 subset after AHST. Of interest, the IFN-γ signaling pathway was the top ranked differential pathway in the ID group. Co-expression network analysis was performed based on combined differential genes in pre-treatment and post-treatment of IF and ID group respectively. As shown in Figure S1, AHST induced a less packed co-expression network in IF group compared to ID group, shown as a significant reduced pattern of connectivity post- than pre-transplantation in IF group. The derivation of co-expression networks based on coefficient correlations of all differently modified genes identified a total of 77 and 52 ‘hub’ genes in the IF and ID groups respectively (Figure 3). The top 20 ‘hub’ genes for each group are summarized in Table S3.

## Discussion

The first nonmyeloablative AHST was performed in a single patient with severe refractory systemic lupus erythematosus in Genoa in 1996, with positive outcome [11]. Since then, a wide spectrum of autoimmune diseases has been treated with AHST. For T1D, although some studies suggested T1D in a bone marrow allograft donor might be transmitted to the recipient; other studies suggested it as secondary responses to GVHD [12]. In addition, animal studies revealed genetic associated NOD mice and streptozocin-induced T1D mice can be reversed by allogeneic [13] and autologous stem cell transplantation [14] respectively. Since both genetic and environmental risk factors are involved in human T1D, either type of stem cell transplantation could possibly benefit T1D patients [15]. In the year of 2007, Dr. Julio C. Voltarelli's group reported the encouraging result that 60% of patients treated with AHST achieved insulin independence and remained controlled for a mean of 31 months [6], [7]. The findings reported here were similar, with 67% of patients remaining non-insulin dependent at the 12-month follow-up (Table 1) and the remarkable increase of C-peptide production suggesting improved β cell function. Although prolonged follow-up is needed, our findings clearly provide additional evidence that nonmyeloablative AHST offers effectiveness in newly diagnosed T1D, especially preservation of β cell function. However, a differential clinical response was identified as three patients were still on an intermediate dose of insulin therapy although the pre-treatment levels of age, BMI, duration time, glucose levels, HbA1c, GADA and C-peptide were similar in the two groups.

Consist with Dr. Julio C. Voltarelli's report [6], the patients in our study maintained persistence of anti-GAD antibodies, suggesting that the conditioning regiment did not fully ablate autoreactive B-cell clones. The fact that GADA was significantly decreased in ID group than IF group confirms that the magnitude of the humoral response and GADA titers are not predictive of beta cell reserve or clinical response [16].

Infection in conditioning period and late infection are a major problem contributing to significant mortality and disease relapse in hematopoietic system malignancies after AHST. In our study, two patients in each group had bacteria or fungus infection during conditioning period while no patient presented late complications, suggesting lack of association of infections with clinical response and AHST may serve as a safe strategy to manage T1D patients.

The pathogenesis of T1D is known to involve a complex interaction of immune cells including CD4^+^ and CD8^+^ T cells, NK cells, B cells, and etc [2], raising the question of how the different patterns of immune reconstitution might contribute to recovery of β cell function. In SLE and systemic sclerosis (SS), AHST induced immunologic self-tolerance by both depletion of autoreactive T cells and restoration of immune regulatory network mediated by CD4^+^CD25^+^T cell; the T cell subpopulation recovered from immune-suppression until approximately one year [17], [18]. In our study, both groups showed a declined number of CD3^+^CD4^+^ and CD3^+^CD8^+^T cell population at 6-month follow-up. Surprisingly, we did not find any significant difference in lymphocyte subpopulations either at diagnosis or at 6-month follow-up even when we evaluated the lymphocyte subpopulation in an independent set of data (Table S5). However, we cannot rule out mechanisms involving lymphocyte subpopulation function that occur independent of total cell number. For example, the average numbers of B cell tended to increase in IF group and decrease in ID group (Table 2 and Table S4). At diagnosis, although the number of CD19 and CD20 positive lymphocyte did not differ in the two groups, but ID group tended to show a higher number. Therefore, with our data it is difficult to exclude the roles of B lymphocyte in the determination of AHST response in T1DM patients.

Gene expression changes in the circulating cells were associated with T1D [19]. We therefore performed extensive transcriptome analysis in PBMCs. As expected, we found common transcripts and pathways grouping all patients independent of their response to treatment, demonstrating a similar mechanistic response to transplantation. Some fraction of these changes might be directly related to a favorable response to treatment. Interestingly, the majority of the immune-related genes were upregulated after transplantation. That may be explained by two possibilities: activation of immune regulatory component or expansion of non-specific immune response or both [17], [19]. In our study, no transcription factors for immune regulatory component such as FoxP3 or cytokine IL10 were significantly changed. This led to speculation that it might be the elimination of the islet targeting autoreactive T cells that improved β cell function. Surely further analysis was required. Indeed, in the IF group we observed more transcriptional modifications, indicating higher sensitivity to AHST.

Top two pathway categories commonly identified in both groups included (1) selective expression of chemokine receptors during T-cell polarization and (2) IL12 and Stat4 dependent signaling pathway in Th1 development. These two pathways indicated that T cell was differentiated to Th1 subset after AHST, which was consistent with a recent study displaying predominant reconstitution of Th1 CD4+T after autologous CD34+ stem cell transplantation in patients with systemic sclerosis [20]. T cell regenerated from thymus after AHST was reported to present a new and diverse TCR repertoire [17]. We therefore speculate that the reconstituted Th1 CD4+T cell in our study may be not reactive to insulin β cell. In the ID group the IFN gamma signaling pathway was found to be the most significantly modified pathway. Given the pro-inflammatory role of IFN-γ, it is possible that activation of this pathway may be responsible for the less favorable outcome in ID group [21].

Additionally, the highly connected ‘hub’ genes, which are thought to play an important role in organizing the behavior of biological modules [22], [23], were found to be associated with insulin dependence. Two of these ‘hub’ genes are worth noting. The first is a T1D susceptible gene, TCF7 [24], which was shown to be a critical transcriptional regulator for memory CD8^+^T cell differentiation and longevity [25]. In our study, AHST markedly decreased the number of connections to TCF7 in IF group, indicating a potential memory T cell decline and naive T cell regeneration after AHST. This is an observation consistent with previous findings [17], [26]. The second involves GZMA, the gene encoding Granzyme A. GZMA connections were found to be markedly increased in the ID group. Granzyme A can promote production of inflammatory cytokines from antigen-presenting cells [27] and induce caspase-independent mitochondrial cell death [28]. Granzyme A is expressed in and surrounding the islets during the development of spontaneous diabetes mellitus in the non-obese diabetic mouse [29]. GZMA up-regulation in our study might contribute to insulin resuming in ID group but certainly requires further investigation. Regardless, the distinct transcriptional events suggest different patterns of immune dysregulation exist in T1D patients.

In summary, we evaluated the clinical and molecular response of nonmyeloablative AHST in nine newly diagnosed T1D patients. AHST improved islet cell function, possibly by elimination of the islet specific autoreactive T cells. T1D patients responded differently to AHST, possibly due to distinct transcriptional events in PBMC. Despite the small patient number and prospective study design, we believe our findings provide important insights for understanding AHST effectiveness and possible molecular clues for optimizing AHST management in T1D patients.

## Supporting Information

Figure S1 **Co-expression network analysis.** Co-expression networks were generated by calculating the coefficient correlation of the combined gene-set in the four data sets: pre-treatment in IF (A), post-treatment in IF (B), pre-treatment in ID (C) and post- treatment in ID group (D). Red, genes with higher expression than pretreatment. Green, genes with lower expression than pretreatment. Straight lines represent positive expression correlation between two genes and plotted line negative correlation. Big circles represent genes which have more correlation with all of the other genes in each network, defined ‘hub’ gene.
(DOC)

Table S1 **Genes dysregulated by HSCT in IF group and ID group.**
(XLS)

Table S2 **Top 20 pathway categories in different groups.**
(DOC)

Table S3 **Rewired genes identified by differential connectivity in IF and ID when treated with AHST.** ^a^gene connectivity in IF group post-treatment; ^b^gene connectivity in IF pre-treatment; ^c^difference of gene connectivity between pre-treatment and post-treatment in IF group; ^d^significance of difference between post-treat and pre-treatment in IF group; ^e^gene connectivity in ID group post-treatment; ^f^gene connectivity in ID pre-treatment; ^g^significance of difference between post-treat and pre-treatment in ID group; ^h^significance of difference between post-treat and pre-treatment in ID group.
(DOC)

Table S4 **Phenotype analysis of lymphocyte subpopulations after hematopoietic stem cell transplantation (AHST) in another set of patients^a^.** ^a^Values are the mean±SD counts/µL. See Results for description of groups; ^b^P value between at diagnosis and 6 months in IF group using paired T test; ^c^P value between at diagnosis and 6 months in ID group using paired T test.
(DOC)

Table S5 **Phenotype analysis of lymphocyte subpopulations at diagnosis and six months after AHST in another set of patients^a^.** ^a^Values are the mean±SD counts/µL. See Results for description of groups; ^b^P value at diagnosis between IF group and ID group using Mann-Whitney test; ^c^P value at six months after AHST between IF group and ID group using Mann-Whitney test.
(DOC)
