# Supplementary material for: Acute Response of Peripheral Blood Cell to Autologous Hematopoietic Stem Cell Transplantation in Type 1 Diabetic Patient
Source: PLoS One. 2012 Feb 22;7(2):e31887. doi: 10.1371/journal.pone.0031887 (PMC3285188; doi:10.1371/journal.pone.0031887)

**Figure Legends**

**Fig S1. Co-expression network analysis.** Co-expression networks were generated by calculating the coefficient correlation of the combined gene-set in the four data sets: pre-treatment in IF (A), post-treatment in IF (B), pre-treatment in ID (C) and post- treatment in ID group (D). Red, genes with higher expression than pretreatment. Green, genes with lower expression than pretreatment. Straight lines represent positive expression correlation between two genes and plotted line negative correlation. Big circles represent genes which have more correlation with all of the other genes in each network, defined ‘ hub’ gene.

Fig. S1


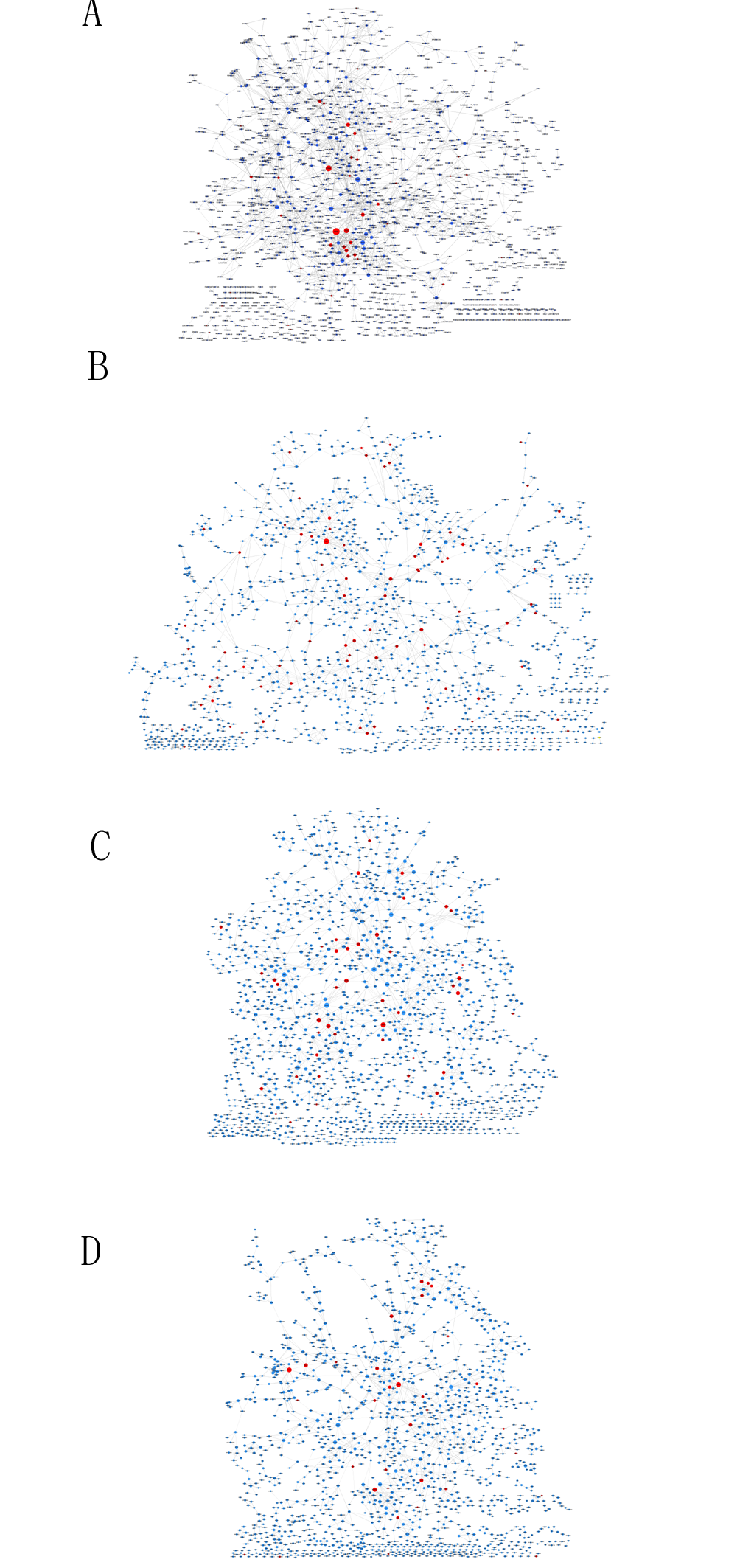

Supplement: Figure S1 — Co-expression network analysis. Co-expression networks were generated by calculating the coefficient correlation of the combined gene-set in the four data sets: pre-treatment in IF (A), post-treatment in IF (B), pre-treatment in ID (C) and post- treatment in ID group (D). Red, genes with higher expression than pretreatment. Green, genes with lower expression than pretreatment. Straight lines represent positive expression correlation between two genes and plotted line negative correlation. Big circles represent genes which have more correlation with all of the other genes in each network, defined ‘hub’ gene. (DOC) [file pone.0031887.s001.doc]
