# Supplementary material for: Acute Response of Peripheral Blood Cell to Autologous Hematopoietic Stem Cell Transplantation in Type 1 Diabetic Patient
Source: PLoS One. 2012 Feb 22;7(2):e31887. doi: 10.1371/journal.pone.0031887 (PMC3285188; doi:10.1371/journal.pone.0031887)
Supplement: Table S2 — Top 20 pathway categories in different groups. (DOC) [file pone.0031887.s003.doc]

**Table S2.** Top 20 pathway categories in different groups.

| Common in both group | | | Specific in IF | | Specific in ID | |
| --- | --- | --- | --- | --- | --- | --- |
| Signal pathway | P value in IF | P value in ID | Signal pathway | P value | Signal pathway | P value |
| Selective expression of chemokine receptors during T-cell polarization | 2.40-E08 | 1.35-E03 | Adhesion and Diapedesis of Lymphocytes | 1.06E-06 | IFN gamma signaling pathway | 2.73-E08 |
| IL12 and Stat4 Dependent Signaling Pathway in Th1 Developmen | 9.10-E07 | 1.65-E03 | Stress Induction of HSP Regulation | 1.03-E05 | TPO Signaling Pathway | 1.46-E04 |
| CTL mediated immune response against target cells | 1.03-E05 | 1.19E-05 | CTCF: First Multivalent Nuclear Factor | 1.03-E05 | Bioactive Peptide Induced Signaling Pathway | 2.90-E04 |
| IL-2 Receptor Beta Chain in T cell Activation | 4.49-E04 | 1.42-E03 | Th1/Th2 Differentiation | 1.35-E05 | IL22 Soluble Receptor Signaling Pathway | 3.76-E04 |
| Mechanism of Gene Regulation by Peroxisome Proliferators via PPARa(alpha) | 5.56-E06 | 1.52-E03 | D4-GDI Signaling Pathway | 4.96-E05 | EGF Signaling Pathway | 5.23-E04 |
| IL-7 Signal Transduction | 3.55-E04 | 2.37-E05 | Adhesion and Diapedesis of Granulocytes | 6.33-E06 | PDGF Signaling Pathway | 6.10-E04 |
|  |  |  | Role of Tob in T-cell activation | 8.26-E05 | Chaperones modulate interferon Signaling Pathway | 7.65-E04 |
|  |  |  | The Co-Stimulatory Signal During T-cell Activation | 9.37-E05 | BCR Signaling Pathway | 9.39-E04 |
|  |  |  | Multiple antiapoptotic pathways from IGF-1R signaling lead to BAD phosphorylation | 9.37-E04 | Ras-Independent pathway in NK cell-mediated cytotoxicity | 9.42-E04 |
|  |  |  | Adhesion Molecules on Lymphocyte | 1.65-E04 | IFN alpha signaling pathway | 1.02-E03 |
|  |  |  | WNT Signaling Pathway | 2.67-E04 | Apoptotic Signaling in Response to DNA Damage | 1.38-E03 |
|  |  |  | Cells and Molecules involved in local acute inflammatory response | 3.55-E04 | IL 6 signaling pathway | 1.38-E03 |
|  |  |  | NO2-dependent IL 12 Pathway in NK cells | 3.55-E04 | Hypoxia and p53 in the Cardiovascular system | 1.65-E03 |
|  |  |  | Transcription factor CREB and its extracellular signals | 3.55-E04 | Granzyme A mediated Apoptosis Pathway | 1.95-E03 |
