# Supplementary material for: Acute Response of Peripheral Blood Cell to Autologous Hematopoietic Stem Cell Transplantation in Type 1 Diabetic Patient
Source: PLoS One. 2012 Feb 22;7(2):e31887. doi: 10.1371/journal.pone.0031887 (PMC3285188; doi:10.1371/journal.pone.0031887)
Supplement: Table S3 — Rewired genes identified by differential connectivity in IF and ID when treated with AHST. agene connectivity in IF group post-treatment; bgene connectivity in IF pre-treatment; cdifference of gene connectivity between pre-treatment and post-treatment in IF group; dsignificance of difference between post-treat and pre-treatment in IF group; egene connectivity in ID group post-treatment; fgene connectivity in ID pre-treatment; gsignificance of difference between post-treat and pre-treatment in ID group; hsignificance of difference between post-treat and pre-treatment in ID group. (DOC) [file pone.0031887.s004.doc]

**Table S3.** Rewired genes identified by differential connectivity in IF and ID when treated with AHST.

| IF group | | | | | ID group | | | | |
| --- | --- | --- | --- | --- | --- | --- | --- | --- | --- |
| Gene symbol | Post-treatment^a^ | Pre-treatment^b^ | Diff K^c^ | P value^d^ | Gene symbol | Post-treatment^e^ | Pre-treatment^f^ | Diff K^g^ | P value^h^ |
| HBA1 | 24 | 2 | 0.93 | 1E-12 | ZNF12 | 17 | 4 | 0.71 | 0.007 |
| FAM153A | 12 | 2 | 0.43 | 0.002 | PRSS23 | 16 | 3 | 0.73 | 0.005 |
| PRSS23 | 11 | 3 | 0.36 | 0.01 | MTERFD2 | 13 | 1 | 0.69 | 0.002 |
| RASSF4 | 10 | 2 | 0.35 | 0.006 | PELI | 12 | 3 | 0.49 | 0.04 |
| SLC25A37 | 10 | 0 | 0.42 | 2E-4 | FRMD3 | 8 | 0 | 0.47 | 0.008 |
| QKI | 9 | 1 | 0.34 | 0.007 | DPH5 | 11 | 2 | 0.50 | 0.02 |
| NFAT1 | 9 | 0 | 0.38 | 0.0005 | PECAM1 | 9 | 1 | 0.46 | 0.02 |
| TRA | 11 | 4 | 0.33 | 0.03 | BACH2 | 10 | 2 | 0.45 | 0.04 |
| CLMN | 10 | 3 | 0.32 | 0.02 | CBX5 | 10 | 2 | 0.45 | 0.04 |
| EIF5 | 9 | 2 | 0.31 | 0.01 | IFRD1 | 10 | 2 | 0.45 | 0.03 |
| USP28 | 11 | 5 | 0.29 | 0.04 | GZMA | 7 | 0 | 0.41 | 0.02 |
| LRRFIP1 | 8 | 2 | 0.27 | 0.02 | MIAT | 8 | 1 | 0.40 | 0.04 |
| NETO2 | 8 | 2 | 0.27 | 0.02 | SPTLC2 | 8 | 1 | 0.40 | 0.04 |
| EPHA1 | 7 | 1 | 0.26 | 0.02 | CD72 | 6 | 0 | 0.35 | 0.03 |
| OSM | 7 | 1 | 0.26 | 0.02 | LOC7284 | 6 | 0 | 0.35 | 0.03 |
| PTPRM | 7 | 1 | 0.26 | 0.02 | MDH1 | 6 | 0 | 0.35 | 0.03 |
| TRAF3IP3 | 7 | 1 | 0.26 | 0.02 | SPATA13 | 6 | 0 | 0.35 | 0.03 |
| ZNF697 | 7 | 1 | 0.26 | 0.02 | TMEM30B | 6 | 0 | 0.35 | 0.03 |
| HERC3 | 0 | 8 | -0.27 | 0.01 | SFI1 | 0 | 6 | -0.43 | 0.01 |
| SLC43A3 | 0 | 8 | -0.27 | 0.01 | TLR5 | 0 | 6 | -0.43 | 0.01 |
| MYEF2 | 1 | 10 | -0.29 | 0.03 | ANKRD55 | 1 | 7 | -0.44 | 0.04 |
| NSUN6 | 0 | 9 | -0.30 | 0.007 | CPD | 1 | 7 | -0.44 | 0.04 |
| PKIA | 2 | 12 | -0.32 | 0.03 | FASLG | 1 | 7 | -0.44 | 0.04 |
| TTC9 | 1 | 11 | -0.33 | 0.02 | SFRS18 | 1 | 7 | -0.44 | 0.04 |
| MMAB | 0 | 10 | -0.33 | 0.004 | TMEM38B | 1 | 7 | -0.44 | 0.04 |
| UTS2 | 2 | 13 | -0.35 | 0.02 | ALDH8A1 | 0 | 7 | -0.50 | 0.007 |
| TCF7 | 2 | 13 | -0.37 | 0.04 | YIPF6 | 0 | 7 | -0.50 | 0.007 |
| PIK3AP1 | 2 | 14 | -0.38 | 0.01 | CXCR6 | 1 | 8 | -0.51 | 0.02 |
| LOXL3 | 1 | 13 | -0.39 | 0.006 | GRN | 1 | 8 | -0.51 | 0.02 |
| ZNF789 | 1 | 13 | -0.39 | 0.006 | RRAS2 | 1 | 8 | -0.51 | 0.02 |
| ACACB | 4 | 17 | -0.40 | 0.03 | LOC439949 | 2 | 9 | -0.53 | 0.04 |
| RAPGEF6 | 3 | 16 | -0.41 | 0.02 | NUP153 | 2 | 9 | -0.53 | 0.04 |
| CDC7 | 1 | 14 | -0.43 | 0.003 | TARP | 2 | 9 | -0.53 | 0.04 |
| FAM26F | 1 | 14 | -0.43 | 0.003 | APLP2 | 1 | 9 | -0.58 | 0.01 |
| PDE7A | 0 | 13 | -0.43 | 0.001 | CSF2RA | 2 | 10 | -0.60 | 0.02 |
| STRBP | 8 | 25 | -0.50 | 0.03 | PPP1R2 | 3 | 12 | -0.68 | 0.02 |
| TLE2 | 1 | 22 | -0.69 | 0.001 | TCF7L2 | 0 | 11 | -0.79 | 0.001 |
| GAS5 | 7 | 30 | -0.71 | 0.003 | GM2A | 3 | 14 | -0.82 | 0.007 |

^a^ gene connectivity in IF group post-treatment; ^b^ gene connectivity in IF pre-treatment; ^c^ difference of gene connectivity between pre-treatment and post-treatment in IF group; ^d^ significance of difference between post-treat and pre-treatment in IF group;  ^e^ gene connectivity in ID group post-treatment; ^f^ gene connectivity in ID pre-treatment; ^g^ significance of difference between post-treat and pre-treatment in ID group; ^h^ significance of difference between post-treat and pre-treatment in ID group.
