# Supplementary material for: Acute Response of Peripheral Blood Cell to Autologous Hematopoietic Stem Cell Transplantation in Type 1 Diabetic Patient
Source: PLoS One. 2012 Feb 22;7(2):e31887. doi: 10.1371/journal.pone.0031887 (PMC3285188; doi:10.1371/journal.pone.0031887)
Supplement: Table S4 — Phenotype analysis of lymphocyte subpopulations after hematopoietic stem cell transplantation (AHST) in another set of patientsa. aValues are the mean±SD counts/µL. See Results for description of groups; bP value between at diagnosis and 6 months in IF group using paired T test; cP value between at diagnosis and 6 months in ID group using paired T test. (DOC) [file pone.0031887.s005.doc]

**Table S4.** Phenotype analysis of lymphocyte subpopulations after hematopoietic stem cell transplantation (AHST) in another set of patients^a^.

|  | IF group (N=8) | | | ID group (N=8) | | |
| --- | --- | --- | --- | --- | --- | --- |
| Lymphocyte population | At diagnosis | six months after AHST | P value^b^ | At diagnosis | six months after AHST | P value^c^ |
| Total | 2276±524 | 1072±326 | 0.002 | 2025±387 | 1069±191 | 0.000 |
| CD3+ | 1882±413 | 671±215 | 0.001 | 1576±234 | 690±150 | 0.000 |
| CD3+CD4+ | 1042±202 | 221±74 | 0.000 | 817±107 | 252±53 | 0.000 |
| CD3+CD8+ | 678±305 | 384±181 | 0.07 | 578±125 | 357±99 | 0.004 |
| CD19+ | 258±146 | 289±135 | 0.59 | 291±162 | 264±129 | 0.49 |
| CD20+ | 259±146 | 298±136 | 0.56 | 275±97 | 233±144 | 0.271 |
| CD3-CD16+  CD56+ | 124±99 | 81±76 | 0.45 | 110±87 | 95±67 | 0.59 |

^a^ Values are the mean±SD counts/μL. See Results for description of groups; ^b^ P value between at diagnosis and 6 months in IF group using paired T test; ^c^ P value between at diagnosis and 6 months in ID group using paired T test.
