# Supplementary material for: Acute Response of Peripheral Blood Cell to Autologous Hematopoietic Stem Cell Transplantation in Type 1 Diabetic Patient
Source: PLoS One. 2012 Feb 22;7(2):e31887. doi: 10.1371/journal.pone.0031887 (PMC3285188; doi:10.1371/journal.pone.0031887)
Supplement: Table S5 — Phenotype analysis of lymphocyte subpopulations at diagnosis and six months after AHST in another set of patientsa. aValues are the mean±SD counts/µL. See Results for description of groups; bP value at diagnosis between IF group and ID group using Mann-Whitney test; cP value at six months after AHST between IF group and ID group using Mann-Whitney test. (DOC) [file pone.0031887.s006.doc]

**Table S5.** Phenotype analysis of lymphocyte subpopulations at diagnosis and six months after AHST in another set of patients^a^.

|  | At diagnosis | | | six months after AHST | | |
| --- | --- | --- | --- | --- | --- | --- |
| Lymphocytepopulation | IF (N=8) | ID (N=8) | P value^b^ | IF (N=8) | ID (N=8) | P value^c^ |
| Total | 2276±524 | 2025±387 | 0.33 | 1072±326 | 1069±191 | 0.72 |
| CD3+ | 1882±413 | 1576±234 | 0.13 | 671±215 | 690±150 | 0.72 |
| CD3+CD4+ | 1042±202 | 817±107 | 0.05 | 221±74 | 252±53 | 0.28 |
| CD3+CD8+ | 678±305 | 578±125 | 0.72 | 384±181 | 357±99 | 0.88 |
| CD19 | 258±146 | 291±162 | 0.85 | 289±135 | 264±129 | 0.72 |
| CD20 | 259±146 | 275±97 | 0.85 | 298±136 | 233±144 | 0.33 |
| CD3-CD16+CD56+ | 124±99 | 110±87 | 0.80 | 81±76 | 95±67 | 0.72 |

^a^ Values are the mean±SD counts/μL. See Results for description of groups; ^b^ P value at diagnosis between IF group and ID group using Mann-Whitney test; ^c^ P value at six months after AHST between IF group and ID group using Mann-Whitney test.
